# YOLOv7-Peach: An Algorithm for Immature Small Yellow Peaches Detection in Complex Natural Environments

**DOI:** 10.3390/s23115096

**Published:** 2023-05-26

**Authors:** Pingzhu Liu, Hua Yin

**Affiliations:** 1 School of Computer and Information Engineering, Jiangxi Agricultura University, Nanchang 330045, China; kevin_ailover@163.com; 2 School of Software, Jiangxi Agricultura University, Nanchang 330045, China

**Keywords:** target detection, attention module, yellow peach, YOLO v7

## Abstract

Using object detection techniques on immature fruits to find out their quantity and position is a crucial step for intelligent orchard management. A yellow peach target detection model (YOLOv7-Peach) based on the improved YOLOv7 was proposed to address the problem of immature yellow peach fruits in natural scenes that are similar in color to the leaves but have small sizes and are easily obscured, leading to low detection accuracy. First, the anchor frame information from the original YOLOv7 model was updated by the K-means clustering algorithm in order to generate anchor frame sizes and proportions suitable for the yellow peach dataset; second, the CA (coordinate attention) module was embedded into the backbone network of YOLOv7 so as to enhance the network’s feature extraction for yellow peaches and to improve the detection accuracy; then, we accelerated the regression convergence process of the prediction box by replacing the object detection regression loss function with EIoU. Finally, the head structure of YOLOv7 added the P2 module for shallow downsampling, and the P5 module for deep downsampling was removed, effectively improving the detection of small targets. Experiments showed that the YOLOv7-Peach model had a 3.5% improvement in mAp (mean average precision) over the original one, much higher than that of SSD, Objectbox, and other target detection models in the YOLO series, and achieved better results under different weather conditions and a detection speed of up to 21 fps, suitable for real-time detection of yellow peaches. This method could provide technical support for yield estimation in the intelligent management of yellow peach orchards and also provide ideas for the real-time and accurate detection of small fruits with near background colors.

## 1. Introduction

Yellow peaches belong to the rose family and are named for the golden color of their flesh. Yellow peach fruits are large and thick, sweet and sour, with a strong flavor, rich in many nutritional elements, and so have a high nutritional and economic value [1]. According to incomplete statistics, at present, the planting area of yellow peaches is about 1.2 million hectares in China, with an annual output of about 600,000 tons, able to meet the market demand for yellow peaches in quantity, making the yellow peach industry gradually change from demanding quantity to demanding quality. In-situ counting of immature yellow peaches at an early stage is an important part of the yellow peach cultivation process and an important measure to improve the quality [2]. Based on the results of the counts and the identification of the location of the yellow peaches, it is convenient for growers to accurately purchase bags and hire workers [3]. Meanwhile, thinning of fruits and planting strategies could also be adjusted based on the actual fruit condition, and buyers could be contacted in advance or ample room could be set aside before the harvest to minimize losses. Overall, the development of algorithms for the accurate detection of unripe yellow peaches and their practical application in production has important implications for yield estimation and orchard management.

At present, the traditional method of in-situ fruit counting relies mainly on manual visual inspection, which is labor-intensive and inefficient and easily influenced by the subjective factors of the observer. With the continuous improvement of computer performance and the widespread application of deep learning technology, fruit detection technology based on computer vision technology has attracted the attention of researchers, of which the YOLO series, as the current advanced target detection algorithm, is widely used in the detection of fruit quantity and position [4]. For example, Hao et al. [5] proposed migration learning by using the COCO (Microsoft Common Objects in Context) dataset to pretrain the model, introducing the mixup data enhancement method and replacing the original backbone network in the YOLOv3 algorithm with the MobilNet-v3 backbone network, which is used in the green-skinned walnut detection method with a mean value of 94.52%. Song Zhongshan et al. [6] proposed to use the dense connectivity mechanism of DenseNet to replace the last three downsampling layers in the feature extraction network Darknet53 in the YOLOv3 network to enhance the feature propagation so as to achieve the reuse of features, and finally the three algorithms recognized green citrus with a mean accuracy of 80.98%. Song Huaibo et al. [7] improved YOLOv5 to achieve the recognition of oil fruits in natural scenes with a mean accuracy of 98.71%. While, Zhang et al. [8] proposed to add a transformer module with an attention mechanism, change the neck structure from the original PAFPN to BIFPN that performs bidirectional weighted fusion, and add a P2 module with shallow downsampling to the head structure, making the improved model have an improved mAp of 0.55:0.95 by 3.77%. Based on related ideas, Lv Jia et al. [9] proposed to add a strip attention module to the backbone network of YOLOv5 to make the model focus more on the striped sleeves of citrus and branches, while in the classroom a semisupervised method is provided as the student model so that the target detection algorithm can use unmarked samples so as to improve the model’s performance and reduce the reliance on marked samples. The mean accuracy of the improved algorithm in the detection of sleeve citrus and branches reached 77.4% and 53.5%, respectively. Xie et al. [10] proposed to add an attention module to YOLOv5, replacing the loss function so as to add a small target detection layer. The improved algorithm increased the mean accuracy by 12.9% on the Litchi dataset. Zhang et al. [11] proposed a fruit counting algorithm based on YOLOx using sample augmentation of a specific real scene. Zhang et al. [12] proposed a new deep neural network called RTSD-Net capable of detecting strawberries in real time, with a 25.93% speedup over YOLOv4-tiny. Qi et al. [13] proposed a deep learning classification architecture for hyperspectral images combining two-dimensional convolutional neutral. The proposed network structure achieves an accuracy of 0.739 on the test dataset. Yu et al. [14] investigated a deep learning-based computer vision approach for field navigation line extraction in five different field scenarios and successfully deployed it on an embedded system that can be integrated into a robot for future automatic navigation. Angelo et al. [15] focused on the detection of phenotypic traits in tomato plants using a YOLOv5-based single-stage detector (standalone or pooled). Wang et al. [16] used a channel pruning algorithm to prune the YOLOv5s model to improve detection efficiency. Marco et al. [17] used the YOLO algorithm for real-time cluster detection and counting of grapes.

All of the studies above were based on the YOLO algorithm, achieved a good level of online in-situ measurement of fruits, and obtained more satisfactory results, fully illustrating the potential of the YOLO algorithm in the field of fruit recognition and detection. However, the methods above were mainly carried out for crops such as litchi, apples, citrus, and tea-oil fruits, in which the monitored objects had significant differences in their morphology, sizes, or colors from the background and it was relatively easy for the model to find the features. Immature yellow peaches, on the other hand, are very different from the study objects above. In terms of color, immature yellow peaches are green in color and difficult to distinguish from the background foliage. In terms of size, immature yellow peaches are generally smaller and more likely to be obscured and thus missed or misdetected, making it difficult for existing models to achieve accurate detection. In order to address the problems of difficult identification of immature yellow peaches close to leaf color, many small targets and partial blurring, and to consider future solutions for agricultural fruit counting, a target detection algorithm based on the improved YOLOv7 was proposed in this study. The main research framework is shown below:

- Anchor frame information for the new YOLOv7 model is generated by K-means clustering algorithm combined with yellow peach data labels.
- The CA (coordinated attention) module is added to the YOLOv7 backbone network for a better extraction of target features from various yellow peaches.
- The original CIoU loss function is replaced with EIoU to accelerate network convergence and improve model accuracy.
- The P2 module for shallow downsampling is added to the head structure of YOLOv7, and the P5 module for deep downsampling is removed, effectively improving the detection of small targets.

Compared to existing detection algorithms, the YOLOv7-Peach model proposed in this study is a superior detection algorithm that can be adapted to the accurate detection of immature yellow peaches under different environmental conditions.

## 2. Data Acquisition and Preprocessing

### 2.1. Data Acquisition

**Figure 1:**
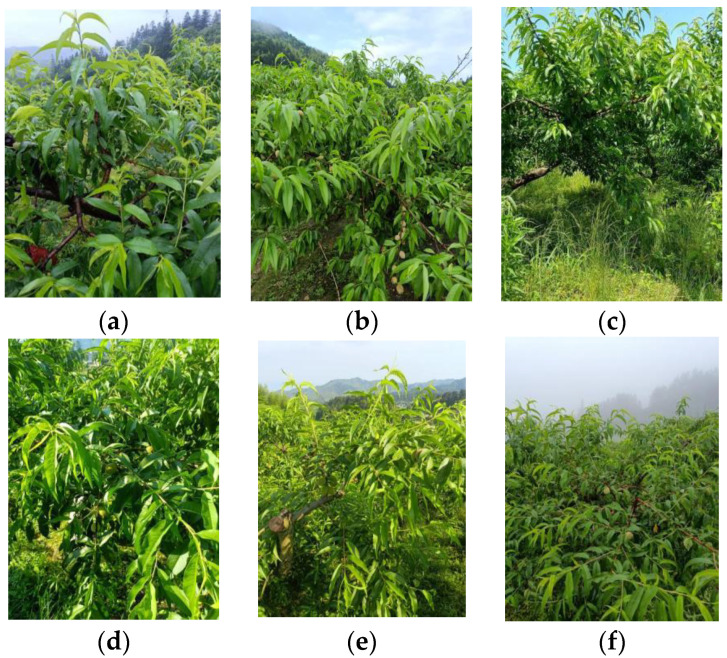
Example of the dataset. (**a**) Rainy, (**b**) Cloudy, (**c**) Sunny, (**d**) Morning, (**e**) Midday, (**f**) Evening.

The data on the mature yellow peaches were collected from the yellow peach plantation in Daping Village, Jinggangshan City, Jiangxi Province, and the photographs were taken between 10 April 2022 and 10 May 2022, when the yellow peaches were already fruiting and growing and about to be bagged. To achieve accurate bagging and fruit thinning management, growers needed to accurately count the number and know the position of yellow peach fruits at this stage. The shooting device was an OPPO A93S mobile phone with a resolution of 4000 pixels by 3000 pixels. To improve the generalization ability of the model, the angle and distance of the shots were not required. After eliminating images that clearly did not meet the requirements (e.g., blurred, shaky), the number of images obtained reached 1021, covering different environments (e.g., morning, midday, evening) and weather conditions (e.g., sunny, cloudy, rainy, etc.). Figure 1 shows the images captured under different environments in the dataset.

### 2.2. Data Annotation and Segmentation

**Figure 2:**
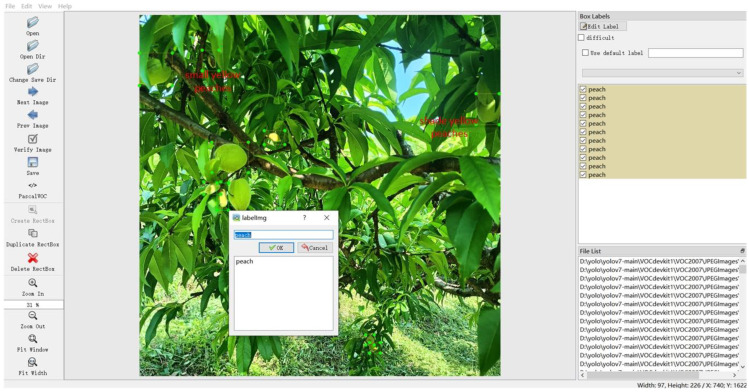
Labeling peach.

The annotation software Labelimg (as shown in Figure 2) was used to annotate the dataset of immature yellow peaches according to the PASCAL VOC 2007 standard. Then, the annotation file was stored in XML format, containing information such as the vertex coordinates of the outer rectangles of the yellow peach targets and the number of instances marked up to 33,743. As the type of dataset used by the YOLO algorithm was txt, the dataset used in the YOLO model was obtained by converting the xml file into a txt format annotation file using python programming language. The dataset was divided into a training set and a validation set in the ratio of 8:2, with 810 and 211 sheets, respectively.

### 2.3. Data Enhancement

**Figure 3:**
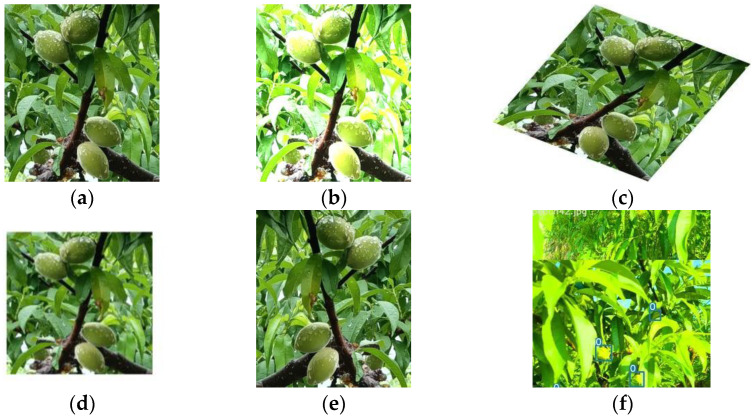
Image augmentation methods. (**a**) Original image, (**b**) Brightness transformation, (**c**) Image rotating, (**d**) Random scaling, (**e**) Horizontal flip, (**f**) Mosaic-4.

To improve the performance of the model and reduce overfitting due to the inadequate size of the dataset, data augmentation must be applied to the original dataset. The methods used included changing brightness, adding noise, random rotation, scaling images, mirroring, and flipping, etc. [18]. In addition, in order to effectively improve the detection accuracy of the model under the perturbation conditions and to increase the diversity of the dataset so as to further improve the generalization ability of the model, the mosaic 4 stitching method [19] was adopted to achieve the data enhancement, in which a portion of each of the four images was intercepted to synthesize a new image and randomly mask parts of the image for overall detection. The details are shown in Figure 3.

## 3. YOLOv7-Peach Detection Model

### 3.1. YOLOv7 Algorithm

**Figure 4:**
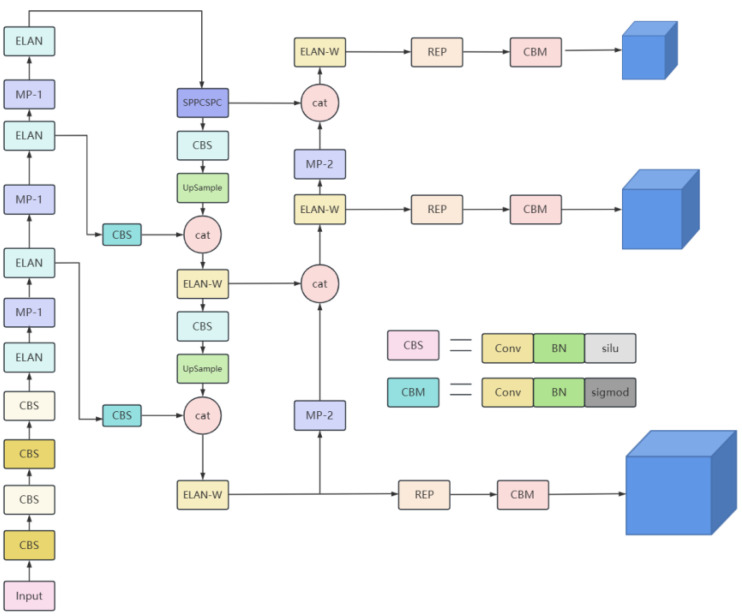
YOLOv7 network architecture.

The YOLOv7 algorithm is one of the more advanced target detection algorithms available. The network structure of YOLOv7 follows the overall layout of the YOLO series, which can be roughly divided into three parts: the spine, the backbone, and the neck. The specific structure is shown in Figure 4. From the input side of the network, images are adaptively scaled, and then the image information is input into the network. The ELAN structure is divided into two branches, the first one going through an 11 convolution for the channel number change, but the second one going through an 11 convolution module for the channel number change first, and then through four 33 convolution modules for the feature extraction, and finally these four features are superimposed together to obtain the final feature extraction result. The MP-1 structure has two branches, the first one going through the MaxPool for a downsampling and then an 11 convolution for the channel number change, but the second one going through an 11 convolution for the channel number change and then through a convolution block with a 33 kernel and a step size of 2 for a downsampling. Finally, the results of the two branches are added together to obtain the final downsampling result. The neck section contains the PANet (Path Aggregation Network) and SPPSCPC (Spatial Pyramid Pooling Cross Stage Partial Concat) modules. Top-down aggregation of the high-layer feature information with the output features of the ELAN module in different layers is conducted, followed by bottom-up aggregation of the shallow-layer features through a bottom-up path aggregation structure, thus fully fusing the image features of different layers. The SPPSCPC (Spatial Pyramid Pooling Cross Stage Partial Concat) module is divided into two branches, the first one changing the channel number through three CBS modules, and then going through four MaxPool branches for processing and tensor splicing, and then changing the channel number through two CBS modules, but the second one going through the CBS module. Finally, the features of the first branch are fused with those of the second one, which then goes through a CBS module for the channel number change [20].

### 3.2. Improved YOLOv7 Algorithm: YOLOv7-Peach

**Figure 5:**
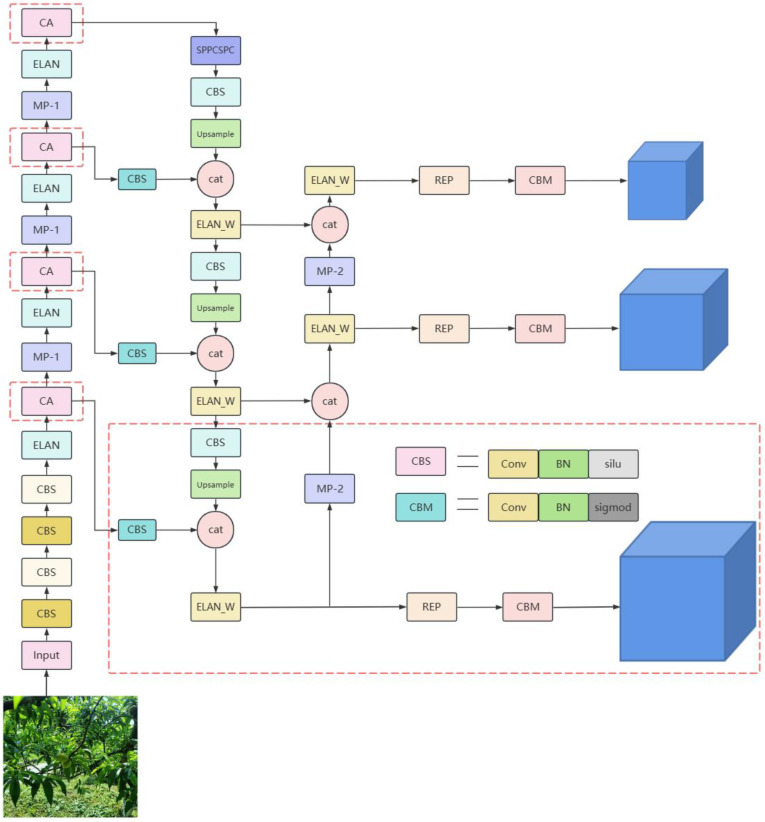
Network structure of the YOLOv7-Peach.

Although the traditional YOLOv7 algorithm is powerful, its detection of immature yellow peaches is unsatisfactory. The missed and wrong detection mainly occurs in the case of small target detection and severe occlusion. Considering this situation, in this paper, we redesigned the anchor frame to suit the characteristics of the yellow peach dataset. The appropriate anchor frame size can quickly match the yellow peach to improve the detection effect and added the CA module to the backbone network, which allows the network to focus on more information about the yellow peach to remove redundant leaf interference information and enhance the feature extraction for the original image. The p2 detection layer for small targets is introduced. The convolutional network is prone to loss of small target information during information transfer, but the addition of the p2 detection layer can retain more small target information and transfer the information deeper into the network to improve the detection effect. The loss function is also replaced with EIoU to speed up the convergence rate, and the improved algorithm is called YOLOv7-Peach, and its structure is shown in Figure 5.

#### 3.2.1. Anchor Redesigning

The initial anchor box size used in the YOLO v7 algorithm was clustered from the object box size in the COCO dataset and many types of items existed in the COCO dataset resulting from the varying sizes of anchor boxes. However, in this dataset, yellow peaches were basically the same size, mainly small- and medium-sized objects, which differed significantly from the object sizes in the COCO dataset. Therefore, the original size of the anchor frame data was not suitable for the dataset of this project. To improve the matching probability between the yellow peach objects and the anchor frames, the K-means clustering algorithm was used to redesign the anchor frame size [21].

The K-means algorithm is a classical clustering algorithm that uses a greedy strategy to form K clusters for all samples through multiple iterations of optimization. K prior frames of different sizes are the K clustering centers. The basic process is described as follows.

The distance d is defined as:


(1) d(box, centroid)=1−IoU(box, centroid)

Here, “*box*” denotes the target box and “*centroid*” denotes the center of clustering.

- K points are randomly selected from the dataset as the centers of the initial clusters, with the centers $C=\{c_{1},\;c_{2},\;\ldots \ldots \;,c_{k}\}$
- For each sample $x_{i}$ in the dataset, the distance to the centroid of each cluster is calculated so as to assign it to the class of the corresponding cluster center if its distance to the centroid of the cluster is the smallest.
- For each category i, the study recalculates the cluster centre $c_{i}=\frac{1}{\left|i\right|}\sum x$ for that category (where |i| is the total number of data in that category).
- Steps 2 and 3 are repeated until the position of the cluster centers no longer changes.

The anchor frame coordinates of all training data were used as the input sample, and k = 9 was set to iterate to obtain the anchor frame sizes of nine clustering centers. The results are shown in Table 1 (as the network was improved by removing the P5 detection layer and then adding the P2 layer, the improved one contained only the P2, P3, and P4 detection layers).

#### 3.2.2. Attention Module

**Figure 6:**
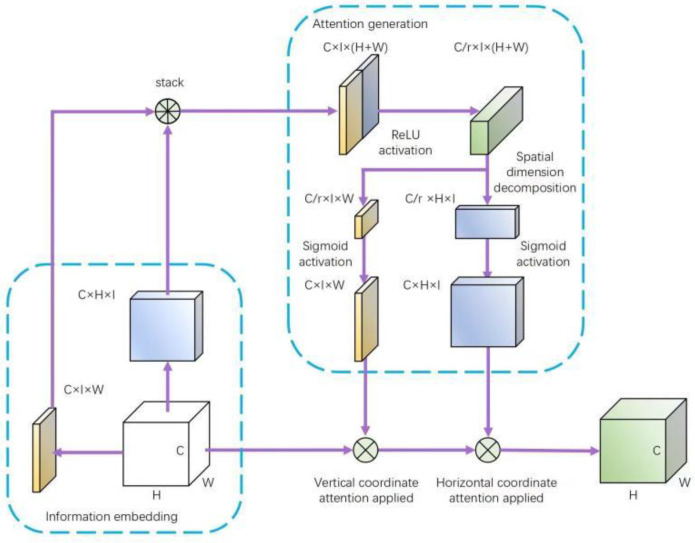
The CA (coordinate attention) module.

The attentional mechanism is a biomimetic visual mechanism that improves the efficiency and accuracy of visual information processing by rapidly scanning the global images, filtering out regions of interest, devoting more attentional resources, and suppressing other useless information [22]. In the natural environment, immature yellow peaches are prone to the problems of fruit overlapping and leaf occlusion, resulting in the loss of the detection accuracy of the model. To address these problems, this paper proposed a coordinate attention mechanism combining location information with channel information and then imposing it in the key positions of the network, as shown in Figure 6, so as to increase the sensitivity of the model to the peach features. Overlapping and occluding targets that were more difficult to identify in the task were assigned high weights so as to increase attention, and low weights were assigned to suppress the uninteresting natural backgrounds so as to improve the recognition accuracy of yellow peaches in the natural environment [23].

#### 3.2.3. Replacement of the Detection Layer

**Figure 7:**
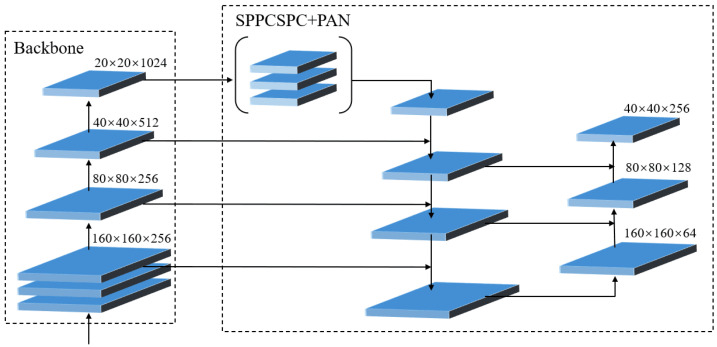
Head network optimization.

In the YOLOv7 algorithm, according to the input features of the head network, the 32, 16, and 8 of the downsampling convolution outputs were selected. The larger the downsampling, the deeper the convolution, and the stronger the semantic representation of the features, which is generally better for the classification but at the cost of more location information loss; whereas, the shallow features with smaller downsampling contained more location information, which was beneficial for the detection of small target objects. Considering the large number of small targets in the yellow peach dataset, this study optimized the head output network as follows: The shallow feature output P2 (four times the downsampling rate) was used as an input feature to the neck network, the large target input feature P5 was removed, and the P3 and P4 features were jointly fused. The neck network used the P2, P3, and P4 as inputs to the head branches, thus improving the detection accuracy of small targets. The detection scales of the original YOLOv7 were (20 × 20 × 255, 40 × 40 × 255, 80 × 80 × 255) and the improved YOLOv7-Peach were (40 × 40 × 255, 80 × 80 × 255, 160 × 160 × 255) [24] (Figure 7).

#### 3.2.4. Loss Function Replacement with EIoU

**Figure 8:**
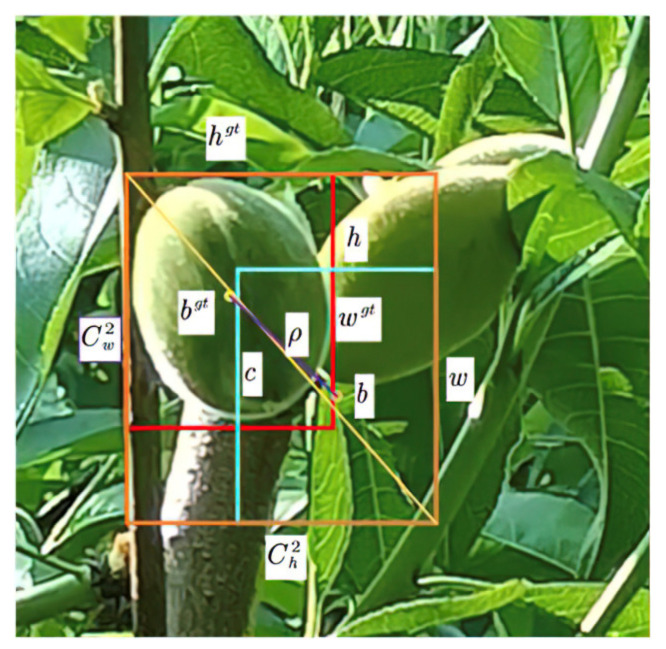
The EIoU loss function graph.

The loss function is the key to the convergence of a deep learning model. Many current target detection algorithms use the joint intersection (IoU) as the loss function for the reason that the intersection ratio presents the error between the prediction frame and the true frame, directly affecting the prediction effect. The higher the value of the loss function, the greater the direct error between the prediction frame and the true frame. However, when there is no intersection between the prediction frame and the true frame, then the loss function does not reflect the problem correctly. To make the prediction frame closer to the true frame and improve the detection effect as well, the original CIoU loss function of the YOLOv7 was replaced, and the efficient joint intersection (EIoU) was used as the loss function in the yellow peach dataset. The basic principle is shown in Figure 8 [25].

In the graph, the red rectangles represent the real frames, the light blue ones represent the prediction frames, and the grey ones are the smallest circular rectangles. $w^{gt}$ represents the width of the real frame, $h^{gt}$ represents the height of the real frame, w represents the width of the predicted frame, and h represents the height of the predicted frame. $b^{gt}$ represents the center of the real frame and b represents the center of the predicted frame. ρ is the distance between the two center positions measured by using Euclidean distance and c represents the distance between the predicted frame and the smallest circular rectangle. The formula is shown below:

(2) $$L_{\mathrm{EIoU}}=L_{\mathrm{IoU}}+L_{\mathrm{dis}}+L_{\mathrm{asp}}\;=1-\mathrm{IoU}+\frac{\rho^{2}(b,\;b^{\mathrm{gt}})}{c^{2}}+\frac{\rho^{2}(w,\;w^{\mathrm{gt}})}{C_{w}^{2}}+\frac{\rho^{2}(h,\;h^{\mathrm{gt}})}{C_{h}^{2}}$$

where $C_{w}^{2}$ and $C_{h}^{2}$ are the width and height of the minimum external matrix of the prediction frame and the GT frame, respectively.

The EIoU divides the loss function into three components: $L_{\mathrm{EIoU}}$ means the loss of the overlapping between the predicted frame and the true frame, $L_{\mathrm{dis}}$ means the loss of the center distance between the predicted frame and the true frame, and $L_{\mathrm{asp}}$ means the loss of the width and the height between the predicted frame and the true frame. The first two components of the EIoU loss continue the approach in the CIoU, while the loss of the width and the height directly minimizes the difference between the width and the height of the predicted frame and the true frame, allowing for a faster convergence.

## 4. Model Training and Evaluation

### 4.1. Experimental Environment and Parameters

All experiments in this paper were performed on a workstation with an AMD(R) EPYC 7351P@2.4 GHz, 16 cores, Nvidia GeForce RTX 3090Ti, and 24 GB of video memory. The operating system was Ubuntu, the programming language was Python 3.8, and the deep learning framework was PyTorch 1.7. The whole training process was set to 300 periods, the batch size of the read-in image data was set to 16, and the input image size was 640 × 640. The initial learning rate was 0.01, and the Adam optimization model was used with a momentum factor of 0.9373.

### 4.2. Evaluation Indicators

The main relevant metrics for evaluating the effectiveness of the neural network models include accuracy, recall, *F*_1_ score [26], PR curve, and average mean accuracy. The accuracy indicates how many samples with positive predictions are actually positive, the recall indicates how many samples with positive predictions are actually positive predictions, and the *F*_1_ score is the summed average of the accuracy and the recall. Generally, the higher the *F*_1_ score, the more stable and robust the model is. AP measures comprehensively the impact of accuracy and recall, and the average AP of all n categories is called the mean average precision (mAp) [27]. The mAp was chosen as the primary model evaluation in the study, comprehensively measuring the accuracy, the recall, and the *F*_1_ scores of the model detection, which are calculated as follows:

(3) $$P=\frac{\mathrm{TP}}{\mathrm{TP}+\mathrm{FP}}$$

(4) $$R=\frac{\mathrm{TP}}{\mathrm{TP}+\mathrm{FN}}$$

(5) $$F_{1}=\frac{2\times P\times R}{P+R}$$

where *TP* indicates the true positive (*TP*), *FP* indicates the false positive (*FP*), *TN* indicates the true negative (*TN*), and *FN* indicates the false negative (*FN*).

## 5. Experimental Results

### 5.1. Ablation Experiments

Ablation experiments were conducted on the YOLOv7-Peach to determine the role of each of the improved modules in the performance improvement of the network, as shown in Table 2. From the results of the ablation experiments, it could be seen that the improved anchor frame size improved the mAp of the network by 0.4%, indicating that redesigning the anchor frame size for the yellow peach target to fit the target detection frame improved the detection accuracy. In addition, when the EIoU loss function was added, the model improved the mAp by 0.5%, which was mainly due to splitting the loss aspects into the difference between the predicted width and height and the minimum external frame width and height, respectively, which accelerated the convergence and improved the regression accuracy. Moreover, the introduction of the FocalLoss optimized the sample imbalance in the bounding box regression task. To be exact, reducing a large number of anchor frames with less overlap with the target frames on the BBox regression caused the regression process to focus on high-quality anchor frames. Additionally, when the CA module was added to the backbone network of the YOLOv7, the mAp was improved by 1.8%, indicating that the attention module could effectively enhance the network for feature extraction and then improve the network accuracy. Replacing the detection head also improved the mAp by 0.8%, which was mainly due to the fact that the p2 detection head was suitable for small target detection with respect to the original network structure, reducing the small target information loss as the network depth increased.

**Figure 9:**
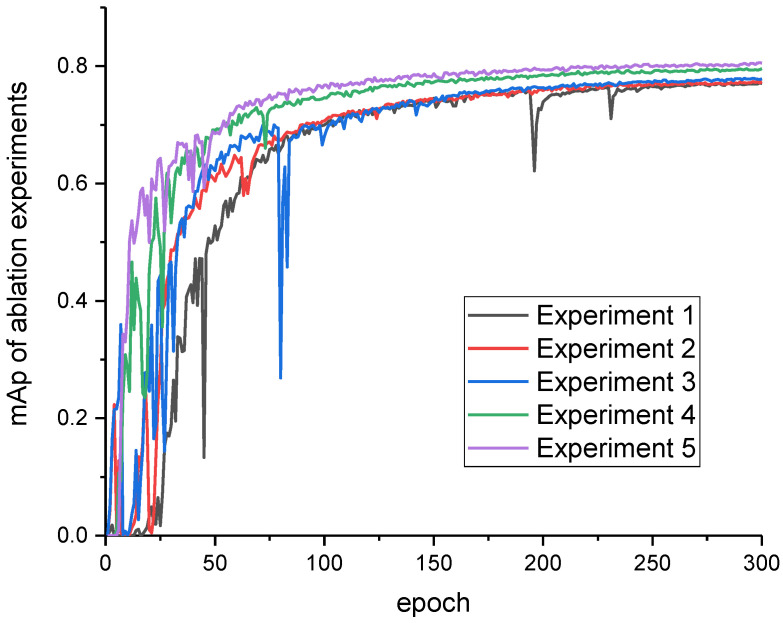
The mAP of ablation experiment.

The accuracy variation of the mAp on the dataset for the ablation experiments and the training variation process are shown in Figure 9. As Figure 9 shows, after reaching one hundred rounds of training, each experiment tended to be smooth. When looking at the variation in the data for Experiments 5 and 4, there was a large fluctuation in Experiment 4 at around 80 rounds, while Experiment 5 had a smaller fluctuation. The addition of the small target detection layer allowed the network to detect objects as small as 4 × 4 pixels, which was a significant improvement for small target detection, and the replacement of the detection layer was better for network training with less variation in the network accuracy. Comparing Experiment 4 with Experiments 1, 2, and 3, it could be seen that the accuracy curve for Experiment 4 was significantly higher than the other curves, indicating that the addition mechanism of the attention module improved the network’s effectiveness significantly, produced better detection results in complex image contexts, and enhanced the extraction of the target information. Observing Experiment 3 and Experiments 1 and 2, the best results were reached faster by adding the EIoU to speed up the convergence in the first 50 rounds or so.

The ablation experiments showed that the mAp of the model improved significantly before and after the improvement. By resetting the anchor frame, using the EIoU as the loss function, adding the attention module to the original model, and improving the detection head, the mAp of the YOLOv7 model for the detection of immature yellow peaches was improved from the original 76.9% to 80.4%, achieving a better result.

### 5.2. Comparison of Different Networks

To demonstrate the benefits of the YOLOv7-Peach model, its performance comparisons were made with other common target detection algorithm models, including the classical target detection network SSD based on target regression (with vgg-16 as the backbone network), other YOLO series, and the latest target detection model ObjectBox [28]. The comparison results are shown in Table 3.

As can be seen from the table, overall, the YOLOv7-Peach was very effective in detecting yellow peaches in terms of both accuracy and recall, with a mAp of 80.4%. The YOLOv7 model did not differ from the YOLOv7-Peach in terms of accuracy but decreased by 3.3% in terms of recalling, indicating that the improved network reduced the occurrence of missed detections in the dataset. In this dataset, the environment of the orchard site was very complex; the leaves in the background were similar in color to the immature yellow peaches, making it very easy to miss the detection. In contrast, the improved network enhanced the recognition of yellow peach features by adding an attention mechanism and replacing the detection head, resulting in a significant increase in the recall. The same was also confirmed in other YOLO series, such as the YOLO v3, v4, and v5 networks, where the recalling ratio was around 0.65, which directly led to their mAp below 0.75. SSD as a classical target detection model, however, in this dataset, despite the high accuracy, the recall ratio was also very low, and so its final MAP was only 54.01%. The latest anchor free-based target detection network, ObjectBox, achieved an accuracy of 83.8% and a recall ratio of 61.4%. However, with a possible extreme imbalance between the positive and negative samples as well as a tendency to cause semantic ambiguity when two target centroids overlapped, the mAp accuracy reached only 69.9%. In practice, we need not only a higher accuracy rate but also a missing rate as low as possible. So, overall, the YOLOv7-Peach is able to balance accuracy and recall rates to meet the project requirements.

As shown in Table 4, the training time, testing time, detection speed, and model size of the model are shown. The training time for the model is 22.5 h, the testing time is 47 ms, the detection speed is 21 fps, and the model size is 51.9 M.

### 5.3. Comparison of Small Target Detection

**Figure 10:**
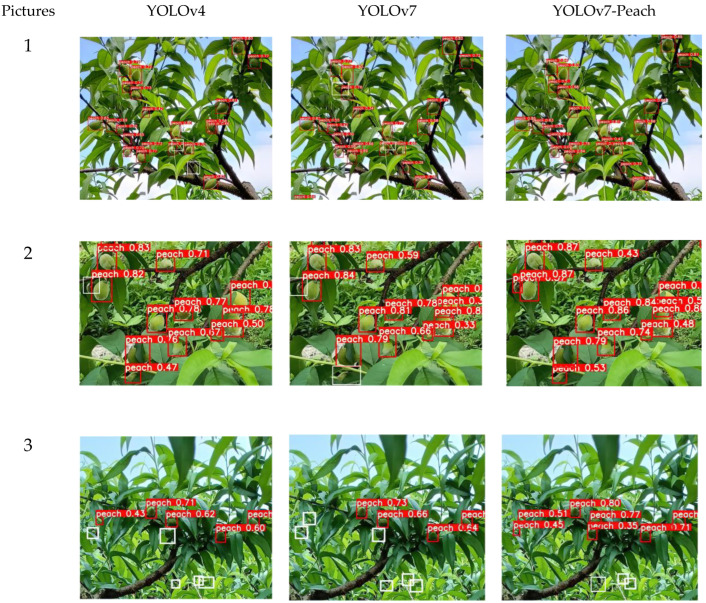
Small target detection (white boxes are missed yellow peaches).

Due to the different camera angles, shooting distances, and growth conditions, various sizes of immature yellow peaches appear in the pictures, and it is very easy to miss the smaller targets when detecting them manually. To verify the advantages of the model, it is necessary to compare the detection of small targets. By comparing different networks, it is well known that the accuracy rates of the YOLOv4 and YOLOv7 are higher than those of other networks. Therefore, the ability of the YOLOv4, YOLOv7, and YOLOv7-Peach to detect small targets is compared, respectively, and the results are shown in Figure 10. 

The true and detected values in the pictures are summarized in Table 5. Combining Figure 9 and Table 4, it can be seen that the YOLOv4 and YOLOv7 algorithms had missed detection on the number region of small targets in Picture 1 (with white boxes indicating the missed detection), two yellow peaches were missed, but the YOLOv7-Peach did not show any missed detection recognition, indicating its significantly better detection ability. In picture 2, both YOLOv4 and YOLOv7 have missed detections, one and two respectively, but YOLOv7-Peach has no missed detections, indicating its better detection effects. All three algorithms in Picture 3 show missed detections, but the YOLOv7-Peach missed fewer, with only three yellow peaches not detected, possibly because the number of the occupied pixel points was too small to be identified, still indicating that the YOLOv7-Peach had stronger detection effects. Smaller targets and denser regions were easily overlooked. The CA module strengthened the attention to the small target information, and even if there were dense regions, the feature information of the region would be noticed and retained, which, to some extent, solved the problem of the missed detection caused by dense overlapping. At the same time, the small target information was more likely to be retained after replacing the detection layer, and less was lost in the information transfer process of the network. Therefore, the YOLOv7-Peach model performed better in the small target detection.

### 5.4. Contrast Test of Occlusion Detection

**Figure 11:**
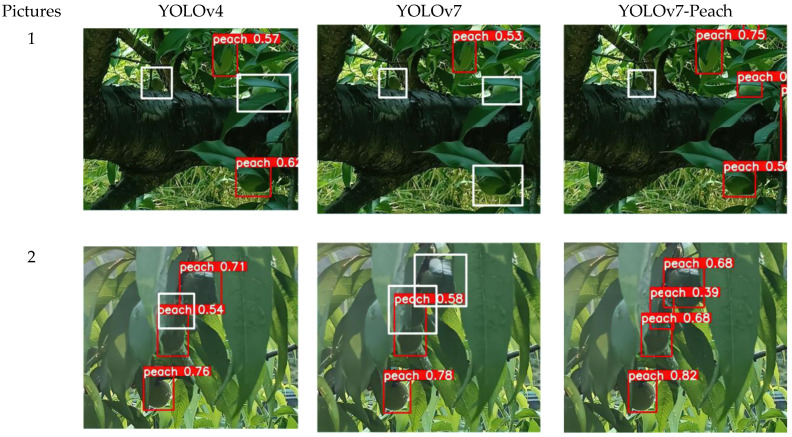

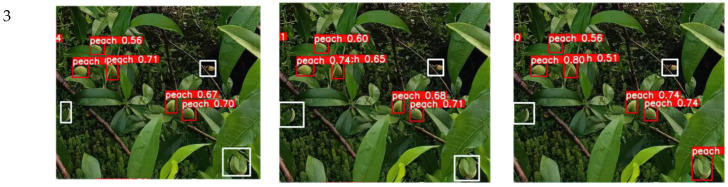
Severe occlusion detection (white boxes are missed yellow peaches).

Usually, the accuracy of the detection algorithm decreases when the target is obscured, especially when the YOLO algorithm is weak in severe occlusion. In the current growth phase, yellow peach trees have denser foliage and the occlusion occurs from time to time. To verify the effectiveness of the YOLOv7-Peach in detecting heavy shading, three heavily shaded images were randomly selected from the test set, and detected by using the YOLOv4, YOLOv7, and YOLOv7-Peach, respectively. The detection effects are shown in Figure 11.

The detection results of the three models for heavily shaded yellow peaches are shown in Table 6. In Pictures 1 and 2 of yellow peaches in the contrast test of occlusion detection, it could be seen that both the original YOLOv7 and YOLOv4 had missed detection of yellow peaches, and the numbers of the missed peaches in both Pictures 1 and 2 (with white boxes indicating the missed detection) were 2 and 1 for both models, respectively. However, the YOLOv7-Peach only appears to miss in Picture 1, indicating its better detection effects. In Picture 3, all three algorithms had missed detection, but the YOLOv7-Peach had fewer missed detections, with only two, while the other two algorithms had three, indicating that the YOLOv7-Peach algorithm was more effective for the occlusion detection. The pictures showed less information about the features of the occluded yellow peaches and lower resolution and further, the convolutional neural network lost more information after multiple passes, resulting in the missed detection caused by severe occlusion. The YOLOv7-Peach introduced the attention module mechanism to enhance the information extraction of the occluded yellow peaches, which focused more on the preservation of the occluded information and lost less information in the process of layer-by-layer information transfer, which helped obtain accurate information about the feature information of yellow peaches in severe occlusion and thus significantly improved the detection efficacy in the case of severe occlusion. The experiments showed that the YOLOv7-Peach with the attention module mechanism could better detect yellow peaches not easily detected in severe occlusion and so achieve better detection results in practice.

### 5.5. Contrast Test of Algorithm Robustness

**Figure 12:**
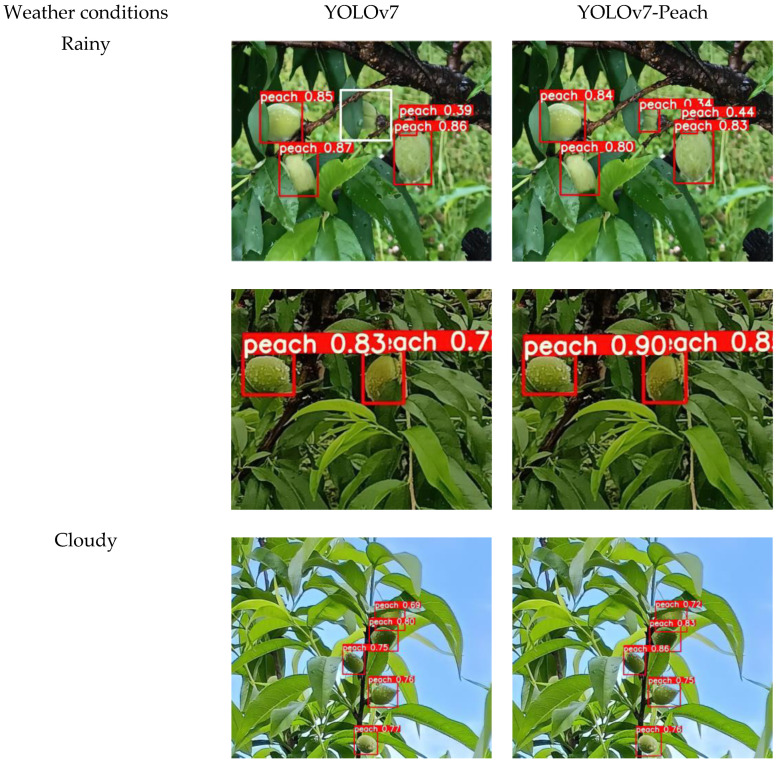

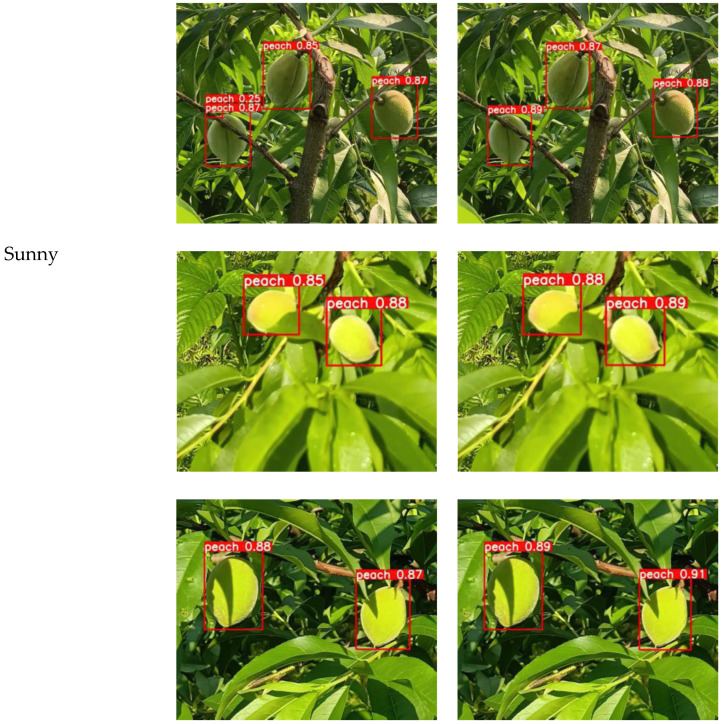
Robustness of YOLOv7-Peach (white boxes are missed yellow peaches).

The actual detection of yellow peaches is prone to interference from various environments, such as weather and lighting conditions, which requires a strong robustness of the algorithm to ensure the accuracy of the detection results in the presence of interference. The algorithm was tested on rainy, cloudy, and sunny days to contrast various detection results of the YOLOv7. The results are shown in Figure 12.

The detection results from under three different weather interferences showed that in the images shot on rainy days, the YOLOv7 had the missed detection, and the number of the missed peach was one, while the YOLOv7-Peach was able to detect all yellow peaches. Under both cloudy and sunny conditions, both algorithms were able to accurately detect yellow peaches, but the confidence level of the results detected by the YOLOv7-Peach was significantly higher than that of the YOLOv7. Different weather conditions and lighting conditions could make the detection difficult, as the original features of the image were altered by the interference, and the features extracted by the convolutional neural network contained a lot of interference information. The feature information of the yellow peaches was affected by noise, so some peaches may not be detected. To cope with this, the robustness of the original algorithm needed to be improved. The YOLOv7-Peach added the CA module to the backbone network to enhance the feature extraction for different types of information, focusing more on important features and weakening interference features. At the same time, the small target detection layer was replaced, reducing the loss of the original information due to the network transfer and focusing more on the small target information. The combination of the two enhanced the detection of the algorithm in an interference environment. Experimentally and theoretically, it is demonstrated that the addition of the CA module and the small target detection layer enhances the robustness of the algorithm and that the YOLOv7-Peach is more suitable for the target detection of yellow peaches in the real-world environment.

### 5.6. Application of Our Method

Based on the proposed algorithm, this paper uses PYQT5 to write a yellow peach detection application, which can complete the detection and quantity count of unripe yellow peaches. The software supports picture input, video input, and real-time camera input, and the program interface is shown in the figure below.

**Figure 13:**
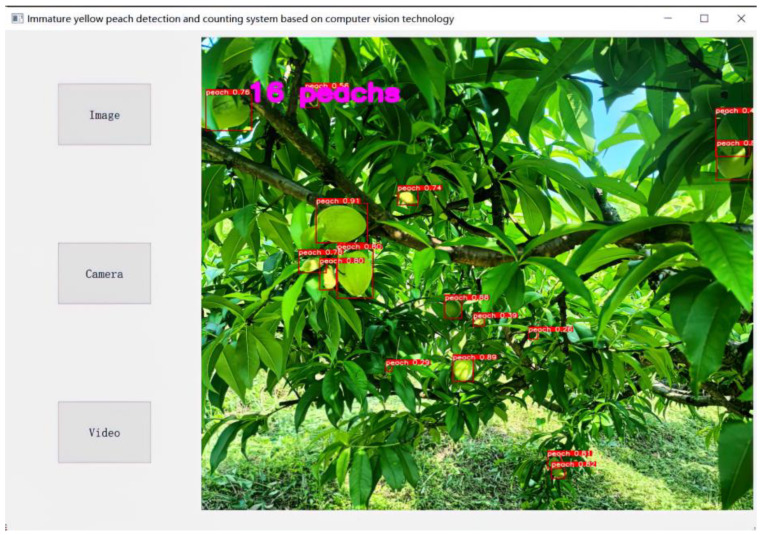
Software interface.

As can be seen from Figure 13, the software identified 16 peaches and the identified yellow peaches varied in form. The algorithm proposed in this paper can meet the task of yellow peach detection and is suitable for promotion in yellow peach orchards.

## 6. Discussion

This paper addressed the problem of difficult detection of yellow peaches in the natural environment and designed the YOLOv7-Peach model based on the improvements of the YOLOv7. The CA module was added to each ELAN module in the backbone network, and also a new loss function EIoU was chosen for the convergence and replacement of the new p2 detection layer. Therefore, the performance of the model in detecting yellow peaches in complex environments was effectively improved. The main conclusions of this paper are stated as follows:

(1) The YOLOv7-Peach algorithm is proposed, which can be used for the yellow peach detection under different complex natural environments. In the ablation experiments, the YOLOv7-Peach algorithm improved the mAp by 3.5%, with an accuracy rate of 79.3%, and improved the recall by 3.3% and the F1 score by 1.8%, as well as the mAp@.5:.95 by 2.5%. It is clear that all evaluation metrics in the improved model worked better than those of the original YOLOv7 network. The YOLOv7-Peach had fewer missed detections and higher accuracy than other models, indicating that the YOLOv7-Peach could provide more reliable support for the yellow peach detection.
(2) The yellow peach dataset for this paper was produced by photographing yellow peaches in a complex natural environment by using various equipment in the natural environment of the yellow peach orchards. The YOLOv7-Peach model was compared with other networks of target detection algorithms, such as the SSD, Objectbox, and YOLO series. The test results showed that the YOLOv7-Peach algorithm achieved good results in terms of the mAp and the recall, reaching 80.4% and 73%, respectively. These two most important metrics were the most effective of the seven different network models.
(3) Although the YOLOv7-Peach model could basically meet the needs of real-time detection in agriculture, the model still had wrong detections and missed detections due to the similar features of leaves and yellow peaches. In view of this, the feature extraction of the input picture information should be strengthened in the subsequent research process to reduce the loss of information caused by the increase in network layers and thus further improve the accuracy of the model.

This project is dedicated to the detection of unripe yellow peaches and in the future, it is hoped that the model can be deployed in hardware. In the meantime, future research will focus on lightweighting the model and improving the speed of detection.

## Figures and Tables

**Table 1:** Comparison of the anchor sizes of the COCO dataset and the yellow peach dataset.

Feature Map Level/Downsampling Multiple	P5/32	P4/16	P3/8	P2/4
	(142, 110)	(36, 75)	(12, 16)	-
COCO anchor size	(192, 243)	(76, 55)	(19, 36)	-
	(459, 401)	(72, 146)	(40, 28)	-
	-	(20, 17)	(10, 13)	(6, 6)
Yellow peach anchor size	-	(21, 25)	(14, 13)	(7, 9)
	-	(33, 39)	(14, 19)	(10, 9)

**Table 2:** Ablation experiments.

Experiment Number	Anchor Redesigning	EIoU	Attention Module	Detection Head Replacement	mAp
1					76.9%
2	√				77.3%
3	√	√			77.8%
4	√	√	√		79.6%
5	√	√	√	√	80.4%

Note: √ means we used this module.

**Table 3:** Network comparison table.

Target Detection Model	mAp	P	R	F1	mAp@.5:95
SSD-VGG	0.5401	0.9332	0.17	0.29	0.225
YOLOv3	0.739	0.82	0.665	0.734	0.37
YOLOv4	0.749	0.813	0.65	0.722	0.364
YOLOv5	0.685	0.787	0.61	0.687	0.312
YOLOv7	0.769	0.793	0.697	0.742	0.371
ObjectBox	0.699	0.838	0.614	0.709	0.339
YOLOv7-Peach(ours)	0.804	0.793	0.73	0.76	0.396

**Table 4:** Other indicators of YOLOv7-peach.

Training Time	Time Spent in Detection (ms)	Detection Speed (FPS)	Size of Model (MB)
22.5 h	47	21	51.9

**Table 5:** Statistics of small target detection results.

Models	Pictures	Real Numbers	Predicted Numbers	Missed Numbers	Average Confidence
	1	22	20	2	0.639
YOLOv4	2	13	12	1	0.653
	3	10	5	5	0.598
	1	22	20	2	0.701
YOLOv7	2	13	11	2	0.661
	3	10	4	6	0.688
	1	22	22	0	0.691
YOLOv7-Peach	2	13	13	0	0.663
	3	10	7	3	0.619

**Table 6:** Statistics of detection results in severe occlusion.

Models	Pictures	Real Numbers	Predicted Numbers	Missed Numbers	Average Confidence
	1	4	2	2	0.595
YOLOv4	2	4	3	1	0.67
	3	8	5	3	0.668
	1	4	1	3	0.530
YOLOv7	2	4	2	2	0.68
	3	8	5	3	0.676
	1	4	3	1	0.5
YOLOv7-Peach	2	4	4	0	0.643
	3	8	6	2	0.693

## Data Availability

Given that the data used in this study were self-collected, the dataset is being further improved. Thus, the dataset is unavailable at present.
